# Application of statistical designs strategy to improve cellulase production using agro-waste residue by a novel isolate *Bacillus licheniformis* strain-MA1 and assessing the enzyme effect on apple juice quality

**DOI:** 10.1186/s12866-024-03656-z

**Published:** 2024-11-29

**Authors:** Mohamed A. A. Abdella, Gamil E. Ibrahim

**Affiliations:** 1 Chemistry of Natural and Microbial Products Department, Pharmaceutical and Drug Industries Research Institute, National Research Centre, Giza, Dokki 12622 Egypt; 2 Chemistry of Flavour and Aroma Department, Food Industries and Nutrition Research Institute, National Research Centre, Giza, Dokki 12622 Egypt

**Keywords:** Cellulase production, Statistical optimization, Apple juice, Antioxidant, Volatile fraction

## Abstract

**Background:**

Cellulose is the major part of lignocellulosic biomass. It can be hydrolyzed into glucose units via specific enzymes called cellulases that have been applied in many commercial fields. There are several studies illustrate the influence of enzymes on apple juice clarification. However, to the best of our knowledge, the effect of microbial cellulase on volatile compounds of apple juice is not well known. The present study aimed to assess the effect of cellulase from a new bacterial isolate on the physicochemical properties of apple juice as well as volatile compounds. The hydrolysis of some polysaccharides (cellulose, hemicellulose, pectin) and polyphenols during apple juice production is necessary to reduce cloud sedimentation or color deterioration and increase the yield of juice. So, enzymes from new microbial isolates serve as processing aids to obtain clear juice with a high yield.

**Results:**

Cellulase-producing bacterium was isolated, characterized and molecularly identified as *Bacillus licheniformis* strain-MA1 with an accession number of ON840115. Optimization of medium parameters was implemented using Plackett–Burman design (PBd) followed by Box-Behnken design (BBd) of response surface methodology (RSM). The PBd revealed the three most important (significant) variables including carboxymethyl cellulose (CMC), corn cob, and peptone that had positive impact on cellulase production. Additionally, using the agricultural residue (corn cob) by the bacterial strain as a carbon source helps in reducing the costs of enzyme production, recycling the by-products, and preserving the environment. The optimized medium using PBd and BBd enhanced cellulase production from *B. licheniformis* strain-MA1 by 6.8-fold. A remarkable increase was observed in juice yield in enzyme treated-juice sample (88.2 ± 0.15%) in comparison with control juice (75.4 ± 0.09%). The total phenolic contents in cloudy and clarified apple juices were 0.957 ± 0.09 and 0.412 ± 0.03 mg/mL, respectively. Also, DPPH and FRAP assays showed a remarkable increase in antioxidant activity (Low IC_50_) in the control sample compared to enzyme treatment. Twenty-seven volatile compounds were extracted using headspace solid-phase microextraction-gas and analysis was performed by GC–MS. The identified volatile constituents belonged to several chemical classes: 15 esters; 6 alcohols; 4 aldehydes and 2 acids. The predominant class in apple juice volatile fraction was esters with a sweet and fruity odor.

**Conclusion:**

The crude cellulase obtained from the novel bacterial isolate *B. licheniformis* strain-MA1 was successfully applied as a clarifying agent in apple juice.

**Supplementary Information:**

The online version contains supplementary material available at 10.1186/s12866-024-03656-z.

## Introduction

Cellulose is a carbohydrate polymer, formed from a linear glucose chain, connected by β−1,4-glucosidic linkages. It is the major part of lignocellulosic biomass and can be hydrolyzed into glucose units via specific enzymes called cellulases [1]. Cellulases are a set of complex biocatalysts, responsible for catalyzing the degradation of cellulose and its related oligosaccharides. The family of cellulase is classified into three major members, endoglucanase or CMCase (EC 3.2.1.4) which randomly hydrolyzes β−1,4-bonds of cellulose chains producing new ends; exoglucanase (EC 3.2.1.91) which acts on cellulose series at the reducing or non-reducing ends generating major products of glucose or cellobiose. The third member in cellulase family is β-glucosidase (EC 3.2.1.21) which can cleave cellobiose and cellodextrins to produce glucose [2–4]. Cellulases can be obtained from several sources like fungi, bacteria, protozoans, animals and plants. Cellulase production has been reported in various bacterial genera including *Bacillus*, *Cellulomonas*, *Clostridium*, *Ruminococcus*, *Paenibacillus*, *Thermomonospora*, *Acetivibrio*, *Erwinia*, *Alteromonas* etc. Among these bacterial genera, *Bacillus* sp. are well recognized as cellulase producers under submerged fermentation such as *Bacillus subtilis*, Bacillus *licheniformis, Bacillus pumilus, Bacillus brevis*, and *Bacillus amyoliquefaciens* [5, 6].

Cellulases have been applied in many commercial fields including detergents, vegetable and fruit juices extraction, textile, pulp and paper, biofuel, brewing, pharmaceutical, food and feed industries [7]. Cellulosic polysaccharides that exist in the fruit cell wall are considered a great hindrance to juice manufacture because they decrease the yield. Also, most of the fibers in fruit juice are cellulosic in nature beside pectin which affects productivity and causes enormous losses to the manufacturers [8]. So, the application of cellulase enzyme is very important for degrading these cellulosic fibers, liquefying fruit pulp, and reducing the viscosity of the final product to obtain better clarification of the juice [8]. As most of the cellulolytic enzymes are of microbial origin, searching for new microbial cellulase producers is very important and might exhibit enormous variance in both enzymatic activity and stability [9]. Optimization of medium nutrients and conditions is considered a fundamental tool for enhancing enzyme production [10]. Statistical methods using Plackett–Burman design (PBd) and response surface methodology (RSM) provide an efficient enhancement approach by studying the combination of various levels of different variables affecting the production process. Moreover, RSM based on Box-Behnken design (BBd) is utilized to optimize the most effective factors and identify their optimal levels to maximize the yield of the enzyme. In addition, the advantage of applying a statistical strategy is to minimize the number of experimental trials required to investigate the interactions between different variables [11]. Obtaining a high juice yield is a critical goal in vegetable and fruit production. The fundamental problem during apple juice production is the presence of some polysaccharides like cellulose, hemicellulose and pectin as well as some polyphenols which make the juice turbid and cloudy and hinder extraction [12]. Hence, the hydrolysis of these polysaccharides and polyphenols is necessary to minimize color deterioration or cloudy sedimentation and maximize the yield of juice. The production of enzymes from new microbial sources has been applied as processing aids to obtain clear juice with higher yield. Apple juice flavour is a decisive criterion to determine the quality and physicochemical properties. The volatile compounds of apple juice belong to several groups like esters, alcohols, aldehydes, and ketones [13, 14]. The main aroma compounds in the apple juice are reported as butyl acetate, ethyl butanoate, (E)−2-hexanal and (E)−2-hexanol which affect by various considerations such as maturity, variety, environmental conditions and processing procedures [15]. Several studies have illustrated the influence of enzymes on apple juice clarification. However, to the best of our knowledge, the effect of microbial cellulase on volatile compounds of apple juice is not well known.

Therefore, the objective of this work was to isolate and identify a new bacterial strain capable of producing cellulase enzyme. Medium parameters were also optimized via multi-factorial designs (PBd followed by BBd) to increase cellulase productivity. Finally, the produced crude cellulase was applied in apple juice clarification with emphasis on juice quality attributes including physicochemical properties, antioxidant activity, phenolic content as well as volatile composition.

## Materials and methods

### Materials

Carboxymethyl cellulose (CMC), yeast extract, peptone, agar and 3,5 dinitrosalicylic acid (DNS) were supplied from Sigma-Aldrich Co. (St. Louis, MO, USA). Furthermore, Magnesium heptahydrate, 1,1′-diphenyl-2-picrylhydrazyl (DPPH), 2,6-di-tert-butyl-4-methylphenol (BHT), Folin-Ciocalteu and gallic acid were purchased from Merck Chemical Company (Darmstadt, Germany). Potassium sodium tartrate, sodium nitrate, calcium chloride and potassium dihydrogen phosphate were purchased from Fluka BioChemica (Buchs, Switzerland). Beef extract, di-potassium hydrogen phosphate and ammonium sulphate were obtained from SDFCL Sd fine-Chem Limited (Mumbai, India). All the other chemicals used were of analytical grade.

### Methods

#### Collection of soil sample

The soil samples were obtained from agricultural lands at Minya El Qamh City, Sharkiya governorate, Egypt (30°27′13.2"N 31°17′55.6"E) and collected in dry, clean bags using a sterile spatula, then transferred to the lab for bacterial isolation. The protocol of this study was approved by the Ethics Committee of the National Research Centre, Cairo, Egypt (approval number: 7444052023).

#### Isolation and qualitative screening for cellulase-producing bacteria

Serial dilutions (from 10^–1^ to 10^–5^) were prepared by suspending 10 g of soil sample in 90 mL of sterile NaCl 0.9% solution. Subsequently, 30 µL from diluted samples were spread on plates containing nutrient agar (NA) medium at pH 7.0 and the cultivated plates were placed in the incubator at 30 °C for 48 h. Finally, the pure bacterial colonies were transmitted to NA slants and kept in the fridge for more investigations [16].

The bacterial isolates were qualitatively tested for their cellulolytic capability by streaking on NA plates supplemented with 1% CMC (carboxymethyl cellulose) as a substrate [6]. After incubation at 30 °C for 48 h, the plates were flooded with Congo red 0.1% (w/v) staining solution, then NaCl solution (1 M) was added to wash off the stain. The positive result for cellulase (CMCase) producer was determined by the appearance of a clear zone surrounding the bacterial isolate [17].

#### Characterization of the cellulolytic bacterial isolate

The selected bacterial isolate (MA1) which can produce cellulase was described morphologically, physiologically, and biochemically to identify it to the genus level based on Bergey’s Manual of Determinative Bacteriology [18]. A series of biochemical tests as well as microscopic investigations and colony features of the isolate MA1 after growing on NA plates for 24 h at 30 °C were observed [16].

#### Molecular identification of the cellulolytic bacterial isolate

For molecular identification, the isolate MA1 was grown in Luria Bertani (LB) broth and incubated at 30 °C for 24 h. Genomic DNA (gDNA) was extracted from the bacterial cells referring to the GeneJET gDNA Purification Kit (#K0721) protocol purchased from Thermo Scientific. By using two universal oligonucleotide primers: 27F (5'-GAGTTTGATCCTGGCTCAG-3') and 1492R (5'- GGTTACCTTGTTACGACTT-3'), Polymerase chain reaction (PCR) amplification of 16S rRNA gene was done via thermocycler (Biometra Thermocycler, Germany). The PCR amplification conditions were started by an incipient denaturation step at 94 °C for 4 min, followed by 35 cycles (94 °C denaturation for 30 s, 55 °C annealing for 30 s, 72 °C extension for 60 s), and final elongation at 72 °C for 10 min. The purified PCR product was sequenced at Macrogen Inc., Seoul, South Korea using an automated DNA sequencer (ABI 3730 XL). The obtained sequence of the 16S rRNA gene was matched with other sequences in NCBI (National Center for Biotechnology Information) using the BLAST algorithm online website (http://www.ncbi.nlm.nih.gov/blast/). A similarity search in the GenBank nucleotides database was performed to confirm the identity of the bacterial isolate and to show its closest phylogenetic relatives [16, 19]. Furthermore, the phylogenetic tree of the isolate MA1 was created by MEGA 11 software (program) using the neighbor-joining method [20].

#### Assessing diverse media for cellulase production by the potent bacterial isolate

For inoculum preparation, nutrient broth containing (g/L): yeast extract, 5.0; peptone, 10.0; NaCl 10.0, was prepared in a conical flask (50 mL, pH 7.0). After sterilization, the flask was inoculated from 24 h old culture of the isolate MA1 and incubated overnight under agitation speed (150 rpm) at 30 °C. Different media (M) were screened for cellulase production by the potent isolate MA1 under submerged fermentation (SmF). These different media (four media) consist of the following components:

- *Medium No.1* (**M1**) composition (g/L): CMC, 10.0; peptone, 5.0; NaNO_3_, 2.0; MgSO_4_, 0.5; K_2_HPO_4_,1.0; FeSO_4_,0.01; KCl, 0.5.0 [21].
- *Medium No.2* (**M2**) composition (g/L): CMC, 3.0; yeast extract, 10.0; (NH_4_)_2_SO_4_, 0.5; MgSO_4_, 0.1; KH_2_PO_4_, 10.0; K_2_HPO_4_, 5.0; NaCl, 0.2 [22].
- *Medium No.3* (**M3)** composition (g/L): CMC, 5.0; peptone, 10.0; beef extract, 5.0; NaCl, 5.0 [23].
- *Medium No.4* (**M4)** composition (g/L): CMC, 10.0; peptone, 10.0; (NH_4_)_2_SO_4_, 1.5; MgSO_4_.7H_2_O, 3.0; KH_2_PO_4_, 2.0; CaCl_2_, 1.0 [24].

The prepared media (50 mL in 250-Erlenmeyer flasks) were adjusted at pH 7.0, sterilized at 121 °C for 20 min, inoculated with one mL of the bacterial inoculum, and then incubated for 48 h at 30 °C in a rotary shaker of 150 rpm. After incubation time, the fermented media were centrifuged at 6,000 × g under cooling (4 °C) for 20 min to get cell-free extract (supernatant) which served as crude cellulase.

#### Cellulase (CMCase) activity assay

The activity of cellulase was investigated as described by Premalatha et al. [9] with some modifications. To start the assay, 0.5 mL of crude cellulase was added to 0.5 mL of 1% CMC prepared in 100 mM sodium phosphate buffer (pH 7.0), then the reaction was placed in a water bath at 40 °C for 30 min. The liberated reducing sugar was determined using the 3,5 dinitrosalicylic acid (DNS) method according to Miller [25] and the developed color was measured by spectrophotometer at 540 nm. One unit (1 U) of enzyme activity was recognized as, the amount of enzyme needed to liberate 1 μmole of reducing sugar (as glucose) per min under assay conditions.

### Cellulase production optimization using multi-factorial designs

#### Plackett–Burman design (PBd)

The PBd was applied to screen a great number of variables having the most important impact on the response [26]. In our work, the impact of eleven independent variables on cellulase production by *B. licheniformis* strain-MA1 was investigated at 2-levels [low (− 1) and high (+ 1)]. These variables included CMC, rice bran, corn cob, peptone, beef extract, (NH_4_)_2_SO_4_, MgSO_4_.7H_2_O, KH_2_PO_4_, CaCl_2_, incubation time, and inoculum volume. Based on PBd, the overall number of experiments was (n + 1) where: n, the number of tested variables so, twelve experimental trials were performed to determine the significant variables and cellulase activity was indicated for response (Table 1). All trials were carried out in triplicates and the mean values were applied for further explanations. The PBd data were fitted with a first-order model based on the following equation:

1 $$\text{Y }= {\upbeta }_{0} +\Sigma {\upbeta }_{\textrm{i}} {\text{X}}_{\textrm{i}}$$

where: Y is the predicted response (cellulase activity U/mL), β_0_ is the model intercept, β_i_ is the linear coefficient (variable estimate) and X_i_ is the independent variable level.

**Table 1:** PBd for screening variables affecting cellulase production by *B. licheniformis* strain-MA1

Trial	A:CMC	B:Rice bran	C:Corn cob	D:Peptone	E:Beef extract	F:(NH_4_)_2_SO_4_	G:MgSO_4_.7H_2_O	H:KH_2_PO_4_	J:CaCl_2_	K:Incubation time	L:Inoculum volume	Cellulase activity	Predicted value
	%	%	%	%	%	%	%	%	%	h	%	U/mL	U/mL
1	(+ 1) 1	(−1) 0	(−1) 0	(−1) 0.2	(+ 1) 0.5	(−1) 0.1	(+ 1) 0.3	(+ 1) 0.2	(−1) 0.05	(+ 1)72	(+ 1) 2	68.7 ± 0.51	71.7
2	(−1) 0.3	(−1) 0	(−1) 0	(+ 1) 1	(−1) 0	(+ 1) 0.3	(+ 1) 0.3	(−1) 0.05	(+ 1) 0.2	(+ 1)72	(+ 1) 2	67.5 ± 0.98	68.5
3	(−1) 0.3	(−1) 0	(−1) 0	(−1) 0.2	(−1) 0	(−1) 0.1	(−1) 0.1	(−1) 0.05	(−1) 0.05	(−1)48	(−1) 1	21.5 ± 0.33	20.4
4	(+ 1) 1	(+ 1) 0.5	(−1) 0	(−1) 0.2	(−1) 0	(+ 1) 0.3	(−1) 0.1	(+ 1) 0.2	(+ 1) 0.2	(−1)48	(+ 1) 2	42.3 ± 1.59	43.3
5	(−1) 0.3	(−1) 0	(+ 1) 0.5	(−1) 0.2	(+ 1) 0.5	(+ 1) 0.3	(−1) 0.1	(+ 1) 0.2	(+ 1) 0.2	(+ 1)72	(−1) 1	35 ± 1.19	31.9
6	(−1) 0.3	(+ 1) 0.5	(+ 1) 0.5	(+ 1) 1	(+ 1) 0.5	(−1) 0.1	(+ 1) 0.3	(+ 1) 0.2	(+ 1) 0.2	(−1)48	(−1) 1	28.9 ± 0.70	27.8
7	(+ 1) 1	(−1) 0	(+ 1) 0.5	(+ 1) 1	(+ 1) 0.5	(−1) 0.1	(−1) 0.1	(−1) 0.05	(+ 1) 0.2	(−1)48	(+ 1) 2	127.6 ± 0.54	130.6
8	(+ 1) 1	(+ 1) 0.5	(+ 1) 0.5	(−1) 0.2	(−1) 0	(−1) 0.1	(+ 1) 0.3	(−1) 0.05	(+ 1) 0.2	(+ 1)72	(−1) 1	111.6 ± 1.50	110.5
9	(−1) 0.3	(+ 1) 0.5	(+ 1) 0.5	(−1) 0.2	(+ 1) 0.5	(+ 1) 0.3	(+ 1) 0.3	(−1) 0.05	(−1) 0.05	(−1)48	(+ 1) 2	46 ± 0.80	47.0
10	(+ 1) 1	(+ 1) 0.5	(−1) 0	(+ 1) 1	(+ 1) 0.5	(+ 1) 0.3	(−1) 0.1	(−1) 0.05	(−1) 0.05	(+ 1)72	(−1) 1	101.8 ± 2.08	98.7
11	(+ 1) 1	(−1) 0	(+ 1) 0.5	(+ 1) 1	(−1) 0	(+ 1) 0.3	(+ 1) 0.3	(+ 1) 0.2	(−1) 0.05	(+ 1)72	(−1) 1	104.9 ± 0.99	101.8
12	(−1) 0.3	(+ 1) 0.5	(+ 1) 0.5	(+ 1) 1	(−1) 0	(−1) 0.1	(−1) 0.1	(+ 1) 0.2	(−1) 0.05	(+ 1)72	(+ 1) 2	27.2 ± 0.21	30.2

#### Box-Behnken design (BBd)

The most significant variables suggested by PBd were further investigated by BBd to obtain the best levels required for maximum enzyme production [27]. According to the results of PBd, three independent variables (CMC, corn cob and peptone) with a positive impact on cellulase production were chosen for further optimization. These variables were studied at 3 levels [low (−1), central (0) and high (+ 1)]. According to BBd, the combinations between the selected variables generated fifteen experimental trials that were executed regarding the mean of cellulase activity as a response (Table 3). The results of BBd were demonstrated using the quadratic model as follows:

2 $$\text{Y }= {\upbeta }_{0} +\Sigma {\upbeta }_{\textrm{i}} {\text{X}}_{\textrm{i}} +\Sigma {\upbeta }_{\textrm{ii}} {\text{X}}_{\textrm{i}}{}^{2} +\Sigma {\upbeta }_{\textrm{ij}} {\text{X}}_{\textrm{i}}{\text{X}}_{\textrm{j}}$$

where: Y is the predicted response (cellulase activity U/mL), β_0_ is the intercept term, β_i_ is the linear coefficient, β_ii_ is the squared coefficient, β_ij_ is the interaction coefficient, X_i_, X_j_ is the independent variables.

#### Software and data analysis

ANOVA involving probability value (*P*-value) and *F*-test was used to identify the effectiveness of the model and independent variables. Also, the efficiency of the model was determined by the regression coefficient (determination coefficient) *R*^*2*^ and the Adjusted *R*^*2*^ [28]. The statistical software Design Expert 13.0 (Stat Ease Inc., Minneapolis, MN, USA) was applied for designing experiments, analysis, and data interpretation.

### Cellulase effect on apple juice quality

#### Fruits and juice processing

The freshly picked large mature apples (*Malus domestica* Var. *Anna*) were purchased from private farms in Sharkiya governorate, Egypt during the season of 2022. The fruits were examined by visual inspection for color, maturity, as well as presence of any infection and by hand press to determine the texture. The fruits with diameters 60–70 mm which had good maturity, color, and were free from any defects or spoiled parts by microorganisms or injury were selected for juice preparation. After that, the fruits were washed with tap water, cut into pieces, and juice was extracted via a suitable juice extractor (Multipress automatic Braun MP80, Germany). Subsequently, the samples were filtered through a four-layered cheesecloth and subjected to enzyme treatment.

#### Apple juice clarification by cellulase

Crude enzyme extract (about 30 U) was incubated with 5 mL of freshly extracted apple juice, filtered through 3 layers of cheesecloth at 50 °C for 1 h and the tube contents were well stirred for mixing the enzyme with juice. The reaction was carried out in a water bath and juice clarification was observed after 50 min. The clarification was evaluated based on physicochemical, rheological and phytochemical analysis as well as volatile compounds according to the method suggested by Pinelo et al. [29].

### Physicochemical analysis

#### Juice yield

The juice yield determined was carried out as mentioned by Vivek et al. [30], using the following equation:

3 $$\text{Juice yield}\ (\%) =\frac{\text{Weight of clear juice}\ (\text{filtrate}) \times 100}{\text{Weight of pulp}}$$

where: Juice yield is the liquid clear juice part obtained from fruit pulp.

#### Measurement of juice stability

Aliquots (10 mL) of the apple juice were drawn from the top of the bottles. Before and after centrifugation at 4200 × *g* for 15 min in a 1 cm path cuvette cell, the cloud stability of the juice was measured as absorbance at 660 nm [31] using UV–Vis Shimadzu Spectrophotometer (UV-1601 PC). The resistance to clarification (turbidity or cloud stability) was deduced from the relative turbidity (*T* %):

4 $$T\;{\%}= (T\text{c}/T\text{o}) \times 100$$

where: *T*o and *T*c are the juice turbidities before and after centrifugation, respectively [32].

#### Rheological measurements

Viscoelasticity of the studied apple juice samples was determined by a rotational type RV (Rheotest 2-Germany) by measuring the time-dependent response of juice subjected to the load in the instrument to be elastic. This characteristic is very important in juice quality due to its effect on the mouthfeel and is used as quality control for juice during storage. The tested samples were introduced into the "S_2_" cylinder of the viscometer. The developed shear stresses at various shear rates (from 1 to 437.4 s^−1)^ as described by Abd-Elrashid et al. [33], were calculated and given with their corresponding curves, from which the slope of the relation between shear stress and shear rate represented the overall apparent viscosity.

The apparent viscosity was calculated according to the following formula:

5 $$\text{Apparent viscoty}\,{\eta}\,={\tau}/{\gamma}\times100$$

where:

η: apparent viscosity in cp

τ: Shear stress (dyn/cm^2^)

γ: Shear rate (sec ^−1^)

(τ) was calculated from the obtained torque value (α) and the cylinder (Z) according to the following equation:

6 $$\uptau =\text{ Z}.\text{ }\alpha$$

## Extraction of phenolic compounds

### Sample preparation

Ten milliliter (10 mL) of apple juice sample was extracted in the absence of light at room temperature with 20 mL of 100% ethyl alcohol containing 1% 2,6-di-*tert*-butyl-4-methylphenol (BHT). The resultant mixture was left at room temperature for 1 h and then filtered through Whatman No. 4 filter paper to exclude protein. By using a rotary evaporator, ethanol was removed from the filtrate, and finally, the deproteinated sample was diluted to the 10 mL original volume [34].

### Determination of total phenolic content (TPC)

The total phenolic content of apple juice samples under investigation was analyzed according to the Folin-Ciocalteu method [35]. The concentrated Folin-Ciocalteu reagent was diluted 10 times with water. Apple juice (0.1 mL) was mixed with 0.75 mL of diluted Folin-Ciocalteu reagent, incubated at room temperature for 5 min, and 0.75 mL of 2% Na_2_CO_3_ solution was added. The mixture was incubated for 15 min at room temperature, and then the absorbance of the solution was measured at 760 nm. The results were expressed as milligrams per liter gallic acid equivalent (GAE) and the calibration curve was carried out with gallic acid aqueous solutions (8–80 ug /mL).

### Determination of antioxidant activity

#### DPPH radical-scavenging spectrophotometric assay

The DPPH (1,1-diphenyl-2-picrylhydrazyl) radical-scavenging activity of juices was determined based on the method of Yen and Chen [36] and 1 mL of the centrifuged juice was diluted with methanol. After that, an aliquot (1 mL) of diluted juice was added to 3 mL of absolute methanol and 1 mL of DPPH solution (0*.*12 g DPPH L^−1^ methanol). The mixture was shaken and left at room temperature for 10 min, and then the absorbance was measured at 515 nm using a UV–Vis Shimadzu Spectrophotometer (UV-1601 PC). The reference cuvette contained DPPH blank. The radical-scavenging activity (antioxidant activity) of the samples was represented as percent inhibition of DPPH radical as follows:

7 $$\%\text{ Inhibition }= [({\text{A}}_{\text{control}} - {\text{A}}_{\text{treatment}} ) / {\text{A}}_{\text{control}} )] \times 100$$

where:

A _control:_ is the absorbance of the control.

A _treatment_: is the absorbance of the treatments.

Percent inhibitions vs. sample volume (uL) curves were applied to determine the concentration at which 50% radical scavenging (IC_50_) occurred [37].

#### Ferric reducing antioxidant power (FRAP)

FRAP assay was done according to the method reported by Benzie and Strain [38]. A total of 300 uL of sample was mixed with 2250 uL of FRAP reagent. Then, the mixture was incubated in the dark at room temperature for 35 min. The absorbance was measured at 593 nm on Shimadzu Spectrophotometer (UV-1601 PC). Percent inhibition vs. sample volume (uL) curves was used to determine the concentration at which 50% radical scavenging (IC_50_) occurred.

#### Headspace Gas Chromatography-Mass Spectrometry (GC–MS) analysis

Headspace-solid phase micro-extraction (HS-SPME) process was performed as described by Kaprasob et al. [39]. Aliquots (10 mL) of juice were added to 3 g NaCl in a 20 mL vial sealed with a PTFE/silicone-coated septum. The volatile compounds were extracted by HS-SPME. The vial was immersed in a water bath at 40 °C for 15 min before fiber exposure. Then, the fiber was exposed to the sample headspace for 60 min at the same temperature under constant stirring, after which GC analysis was conducted. At the injector port, the fiber was desorbed at 250 °C for 10 min and set in splitless mode for 2 min. The compounds were separated on a ZB-WAX Plus polar phase capillary column (Zebron, Phenomenex, USA; 60 m × 0.25 mm × 0.25 μm). Helium was utilized as carrier gas with an initial flow of (0.8 mL min^−1^). The GC oven temperature program was (40 °C for 2.5 min, 5 °C/min ramp to 220 °C, 10 °C/min ramp to 240 °C and holding for 1.5 min) and the temperature in the detector was kept at 230 °C. A series of homologous *n*-alkanes were analyzed under the same conditions to calculate the linear retention index (LRI) for analyte identification. Electron ionization at 70 eV was used to obtain the mass spectra with a scanning range of 35–400 m/z. The mass spectrometry ion source and MS quadrupole temperature were set at 230 °C and 150 °C, respectively. The volatile compounds identification was carried out by comparing experimental mass spectra with those from the National Institute of Standards and Technology library (NIST 05) and the experimental linear retention index (LRI). The obtained values were compared with those in the literature [40].

## Results and discussion

### Isolation and qualitative screening for cellulolytic bacteria

The bacterial isolation exhibited fifteen pure colonies that were screened for cellulolytic activity by cultivation on NA plates containing 1% CMC. The results revealed that one bacterial isolate (MA1) could produce cellulase (CMCase) causing degradation of CMC that was recognized by the presence of a clear area around the colony when staining with Congo red (0.1%) solution and de-staining with NaCl (1 M) solution (Supplementary Fig. S1). The isolate MA1 was cultured on NA slants, preserved at 4 °C, and chosen for further study.

### Characteristics of the cellulolytic bacterial isolate

The bacterial isolate MA1 that showed cellulolytic activity was described on the basis of morphological, physiological and biochemical characteristics [18]. The results indicated that the isolate MA1 was aerobic, short rods, motile, spore-forming and Gram-positive bacteria. Also, the isolate MA1 gave positive results (+) towards several biochemical tests (catalase, urease activity, citrate utilization, nitrate reductase, oxidase activity, methyl red, hydrolysis of casein, CMC, starch, and gelatin, fermentation of glucose, lactose, and sucrose), while it showed negative results (-) towards indole test, hydrolysis of locust bean gum (LBG), xylan, and pectin.

### Molecular identification of the cellulolytic bacterial isolate

**Fig. 1:**
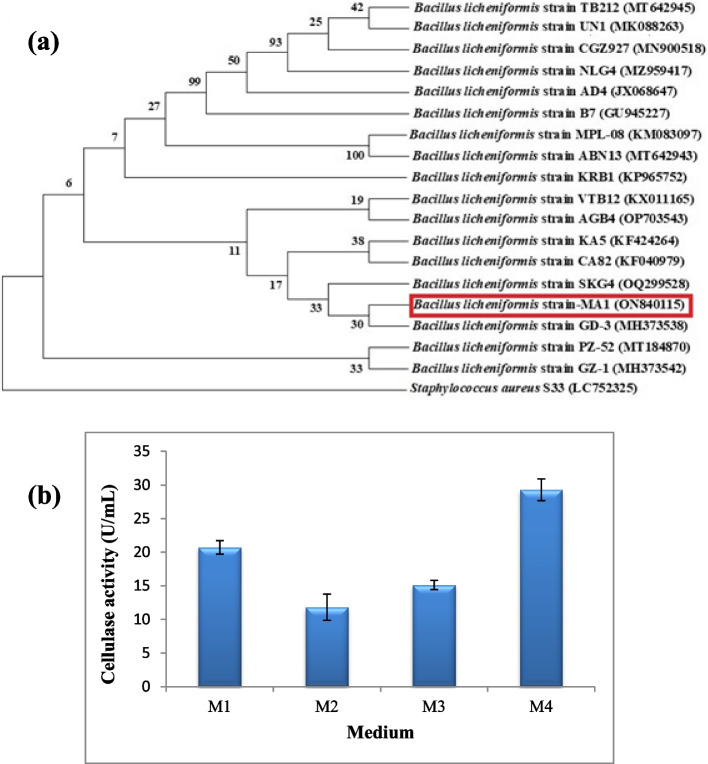
**a **Phylogenetic tree of *B. licheniformis* strain-MA1 (ON840115) depends on the alignment of 16S rRNA gene with some closest phylogenetic neighbors in GenBank. *Staphylococcus aureus* S33 (LC752325) was used as an out-group of the tree. Bootstrap values expressed as a percentage of 500 replications, are given at the branching point. The scale bar represents 5% sequence divergence (**b**) Assessing of diverse media for cellulase production by *B. licheniformis* strain-MA1

The selected isolate MA1 was molecularly identified through sequencing of 16S rRNA gene and phylogenetic analysis. The target gene (16S rRNA) sequence of the isolate MA1 was registered in the NCBI (GenBank) nucleotide database as *Bacillus licheniformis* strain-MA1 under accession number of ON840115. The homogeneity searches for *B. licheniformis* strain-MA1 showed 99% identity with other phylogenetic neighbors in the NCBI. Also, the constructed phylogenetic tree of *B. licheniformis* strain-MA1 (ON840115) with the closest phylogenetic relatives using MEGA 11 software is shown in Fig. 1a.

### Assessing of different media for cellulase production

*B. licheniformis* strain-MA1 was cultivated in diverse media to select the best one for cellulase production. According to the results, the greatest production (29.3 ± 1.33 U/mL) was observed by growing the bacterial strain in M4 which was 2.5 times greater than M2 which showed the lowest productivity (11.8 ± 1.6 U/mL). In addition, when the bacterial strain was grown in M1 and M3, the enzyme yield was (20.7 ± 0.81 and 15.1 ± 0.57 U/mL), respectively (Fig. 1b). The diversity of cellulase production might be referred to the presence of different levels of various metal ions that manipulate microbial growth and enzyme synthesis [16]. So, M4 was used as the basic (control) medium for further production optimization study.

### Multi-factorial designs for optimizing cellulase production

#### Plackett–Burman design (PBd)

The PBd is considered a prominent procedure to choose the most significant factors required to improve cellulase production by *B. licheniformis* strain-MA1 [26]. It provides the evaluation of eleven independent variables that were tested at 2-levels (low and high). Based on the model design, a group of 12 trials and their corresponding response (cellulase activity U/mL) were illustrated in Table 1. Subsequently, a wide range in cellulase activity (from 21.5 ± 0.33 to 127.6 ± 0.54 U/mL) was observed due to various incorporations between various variables levels. According to the results, the greatest enzyme productivity was recorded in trial 7 which exhibited cellulase activity of 127.6 ± 0.54 U/mL.

Also, the relation between cellulase activity and variables was determined by applying multiple- regression analysis of PBd results. The obtained response (Y) for predicted cellulase activity (U/mL) was expressed in terms of linear regression model using the following first-order equation:

8 $$Y = 65.25 +27.57A -5.62B +10.13C +11.07D +2.75E +6.02G -14.08H +3.57J +3.38K$$

where, Y: cellulase activity U/mL (predicted response); A, B, C, D, E, G, H, J, and K: the codes for CMC, rice bran, corn cob, peptone, beef extract, MgSO_4_.7H_2_O, KH_2_PO_4_, CaCl_2_ and incubation time, respectively.

The PBd model significance was affirmed by ANOVA for cellulase production. As illustrated in Table 2, *F*-value (55.52) and probability value (*P*-value of 0.0178) of the model indicate that it was significant. Also, the low p-value of the model terms (*P*-value < 0.05) points to the prominence of the independent variables and their effect on the response. From ANOVA results, four variables including CMC, corn cob, peptone and KH_2_PO_4_ showed a significant impact on cellulase production. On the other hand, rice bran, beef extract, (NH_4_)_2_SO_4_, MgSO_4_.7H_2_O, CaCl_2_, incubation time, and inoculum volume were considered non-significant variables. Furthermore, the goodness and efficiency of the statistical model can be confirmed with the determination coefficient (*R*^*2*^) value. So, the model that exhibits a high *R*^*2*^-value (more than 0.9) has a great correlation between predicted and observed responses [16, 41]. Based on PBd analysis, the *R*^*2*^-value was 0.9960 indicating that 99.60% of the total variance in cellulase production can be interpreted by the statistical model and only 0.4% of the variance is not interpreted. In addition, the Adjusted *R*^*2*^-value (0.9781) points to the significance of the model and confirm the good agreement between the actual and the predicted results. The coefficient of variation (CV) value refers to the degree of accuracy and reliability of the experiments [42]. In this case, the CV value of 8.51% implies that, the model was effective and had a high precision to the experiments.

**Table 2:** ANOVA for PBd of cellulase productivity by *B. licheniformis* strain-MA1

Source	Sum of Squares	DF	Mean Square	Std. Dev	*F*-value	*P*-value	
Model	15,394.74	9	1710.53	5.55	55.52	0.0178	Significant
A-CMC	5401.76	1	5401.76	0.3656	296.01	0.0034	
B-Rice bran	2920.32	1	2920.32	0.2611	12.29	0.0726	
C-Corn cob	34,992.00	1	34,992.00	0.2611	40.00	0.0241	
D-Peptone	3902.41	1	3902.41	0.4178	47.71	0.0203	
E-Beef extract	1012.00	1	1012.00	0.2611	2.95	0.2282	
G-MgSO_4_.7HO	11,681.28	1	11,681.28	0.1044	14.10	0.0642	
H-KH_2_PO_4_	13,790.52	1	13,790.52	0.0783	77.26	0.0127	
J-CaCl_2_	2096.16	1	2096.16	0.0783	4.96	0.1559	
K-Incubation time	745.76	1	745.76	12.53	4.46	0.1691	
Residual	61.61	2	30.81				
Cor Total	15,456.35	11					

*R*^*2*^ = 0.9960, Adjusted *R*^*2*^ = 0.9781, CV = 8.51%, Adequate Precision = 21.743, Std. Dev. (standard deviation),

DF (degree of freedom), Significant (*P* < 0.05), Un-significant (*P* > 0.05)

**Fig. 2:**
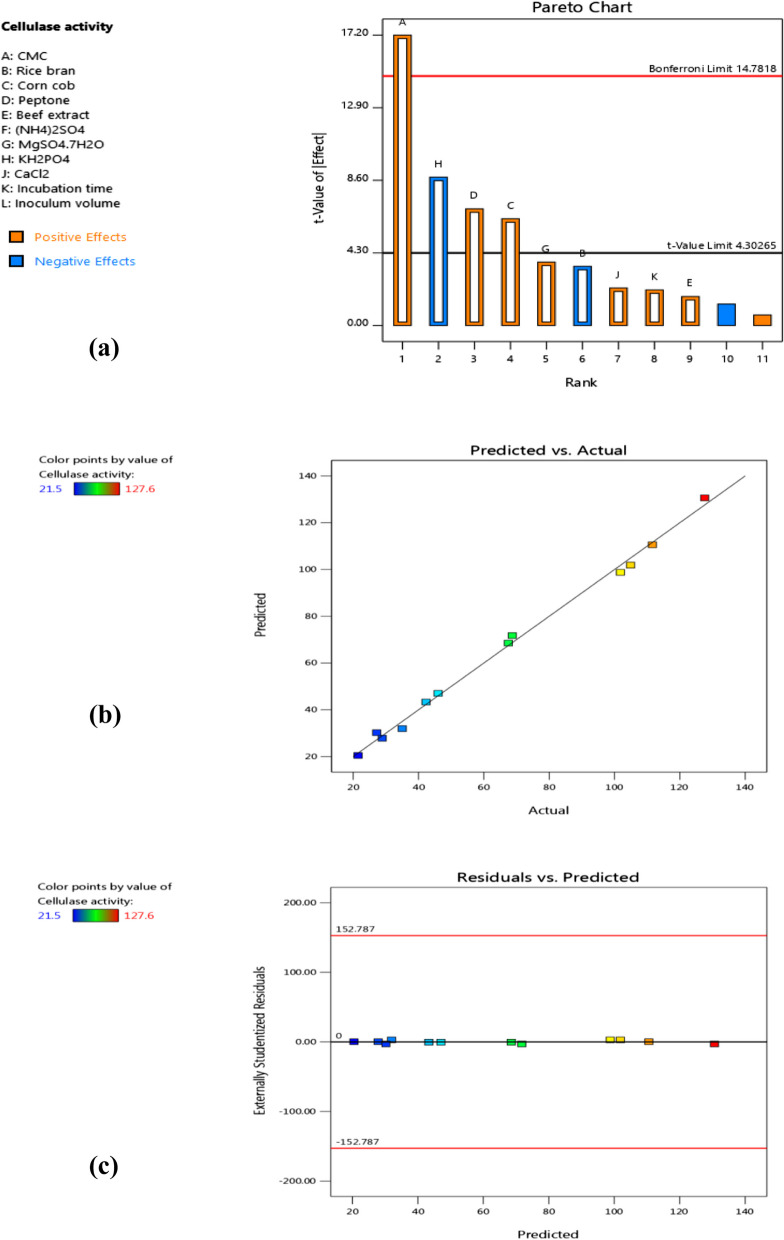
PBd for cellulase production by *B. licheniformis* strain-MA1 (**a**) Pareto chart for displaying significant variables (**b**) The relationship between predicted and actual values (**c**) Chart of residuals vs predicted values

On the other side, the absolute relative significance of PBd is best demonstrated by Pareto chart that displays all variables affecting cellulase activity in descending order. As shown in Fig. 2a, the most significant variables were CMC, corn cob, and peptone that affect cellulase production positively, whereas KH_2_PO_4_ had negative significant impact. Our finding is in consistency with Singh et al. [43] who suggested that, CMC and peptone showed significant effect, while MgSO_4_.7H_2_O showed non-significant effect on cellulase production by *B. amyloliquefaciens* SS35. Also, Shajahan et al. [44] found that, CMC exhibited positive impact on cellulase production from *B. licheniformis* NCIM 5556. On the other hand, (NH_4_)_2_SO_4_ and KH_2_PO_4_ were considered significant variables for cellulase production by *Enhydrobacter* sp.ACCA2 [9]. The relation among actual (observed) and predicted values of cellulase activity was presented in Fig. 2b. The extremely closure between observed and predicted values proves that, the model was effective and able to illustrate enzyme productivity by *B. licheniformis* strain-MA1. Moreover, the chart of residuals vs predicted values shows spreading the residuals about a horizontal line (zero reference) in a random manner providing a good fitness of the regression model to the experimental results as seen in Fig. 2c.

#### Box-Behnken design (BBd)

The next step in enzyme production optimization was carried out using BBd to obtain the ideal values (local best levels) of the most important variables screened by PBd for maximizing cellulase productivity from *B. licheniformis* strain-MA1 [27]. The investigation of the chosen variables (CMC, corn cob and peptone) at 3 different levels (low, central and high) based on BBd matrix provided 15 trials and their corresponding response results were introduced in Table 3. A quadratic model was constructed by implementing multi-regression analyses to the experimental results, which fitted to the following equation:

**Table 3:** BBd to optimize variables affecting *B. licheniformis* strain-MA1 cellulase production

Trial	A: CMC	B: Corn cob	C: Peptone	Cellulase activity	Predicted values
	%	%	%	U/mL	U/mL
1	(−1) 1	(+ 1) 1	(0) 1.5	114.1 ± 0.79	112.33
2	(−1) 1	(0) 0.75	(+ 1) 2	89 ± 0.46	89.88
3	(0) 1.5	(+ 1) 1	(−1) 1	156.4 ± 0.66	154.70
4	(+ 1) 2	(0) 0.75	(+ 1) 2	147.2 ± 0.62	143.72
5	(−1) 1	(0) 0.75	(−1) 1	100.6 ± 0.94	104.08
6	(+ 1) 2	(−1) 0.5	(0) 1.5	178.5 ± 0.33	180.28
7	(0) 1.5	(0) 0.75	(0) 1.5	131.9 ± 0.90	131.80
8	(0) 1.5	(+ 1) 1	(+ 1) 2	119 ± 0.49	119.90
9	(−1) 1	(−1) 0.5	(0) 1.5	67.7 ± 0.65	65.13
10	(0) 1.5	(−1) 0.5	(+ 1) 2	121.5 ± 0.17	123.20
11	(0) 1.5	(0) 0.75	(0) 1.5	129.4 ± 1.10	131.80
12	(+ 1) 2	(0) 0.75	(−1) 1	190.2 ± 0.31	189.33
13	(0) 1.5	(0) 0.75	(0) 1.5	134.1 ± 0.54	131.80
14	(+ 1) 2	(+ 1) 1	(0) 1.5	133.7 ± 0.75	136.28
15	(0) 1.5	(−1) 0.5	(−1) 1	149.1 ± 0.38	148.20

9 $$Y=131.80+34.77A-7.93B-14.95C-22.80AB-7.85AC-2.45BC-6.52A^{2}-1.770B^{2}+6.47C^{2}$$

Where, Y: cellulase activity U/mL (predicted response); A, B and C: the codes for CMC, corn cob, and peptone, respectively.

The strength of BBd and each term in the second-order model were determined via ANOVA for cellulase activity (Table 4). From the results, BBd exhibited significant impact based on its *F*-value (123.55) and *P*-value < 0.05. The data refers to a great variance in response which can be demonstrated through the equation of the regression model. Furthermore, the p-values of the model terms including linear (A, B, C), interactions (AB, AC), and quadratic (A^2^, C^2^) imply that these terms were significant. Also, the CV value was very low (2.73%) that implies the high degree of reliability for the experimental results. On the other hand, the lack of fit was not significant, that indicates the model had a perfect convenience and adequate precision to the experiments. Moreover, the accuracy of BBd was achieved by the *R*^*2*^ which had value of 0.9955 that means 99.55% of the total distinction in cellulase activity can be elucidated by the model and only 0.45% of these distinctions cannot be elucidated. Besides, the Adjusted *R*^*2*^-value (0.9875) and the Predicted *R*^*2*^-value (0.9391) refer to the great significance of the statistical model. So, BBd confers a good elucidation about the relation between the tested variables and cellulase production by *B. licheniformis* strain-MA1.

**Table 4:** ANOVA of BBd for optimizing cellulase production by *B. licheniformis* strain-MA1

Source	Sum of Squares	DF	Mean Square	Std. Dev	*F*-value	*P*-value	
Model	14,167.12	9	1574.12	3.57	123.55	< 0.0001	Significant
A-CMC	9674.40	1	9674.40	0.3780	759.31	< 0.0001	
B-Corn cob	502.44	1	502.44	0.1890	8.93	0.0305	
C-Peptone	1788.02	1	1788.02	0.3780	140.34	< 0.0001	
AB	2079.36	1	2079.36		163.20	< 0.0001	
AC	246.49	1	246.49		19.35	0.0070	
BC	24.01	1	24.01		1.88	0.2282	
A^2^	157.20	1	157.20		12.34	0.0171	
B^2^	11.63	1	11.63		0.9130	0.3832	
C^2^	154.80	1	154.80		12.15	0.0176	
Residual	63.71	5	12.74				
Lack of Fit	52.65	3	17.55		3.17	0.2488	Un-significant
Pure Error	11.06	2	5.53				
Cor Total	14,230.83	14					

*R*^*2*^ = 0.9955, Adjusted *R*^*2*^ = 0.9875, Predicted *R*^*2*^ = 0.9391, CV = 2.73%, Adequate Precision = 42.615

Std. Dev. (standard deviation), DF (degree of freedom), Significant (*P* < 0.05), Un-significant (*P* > 0.05)

**Fig. 3:**
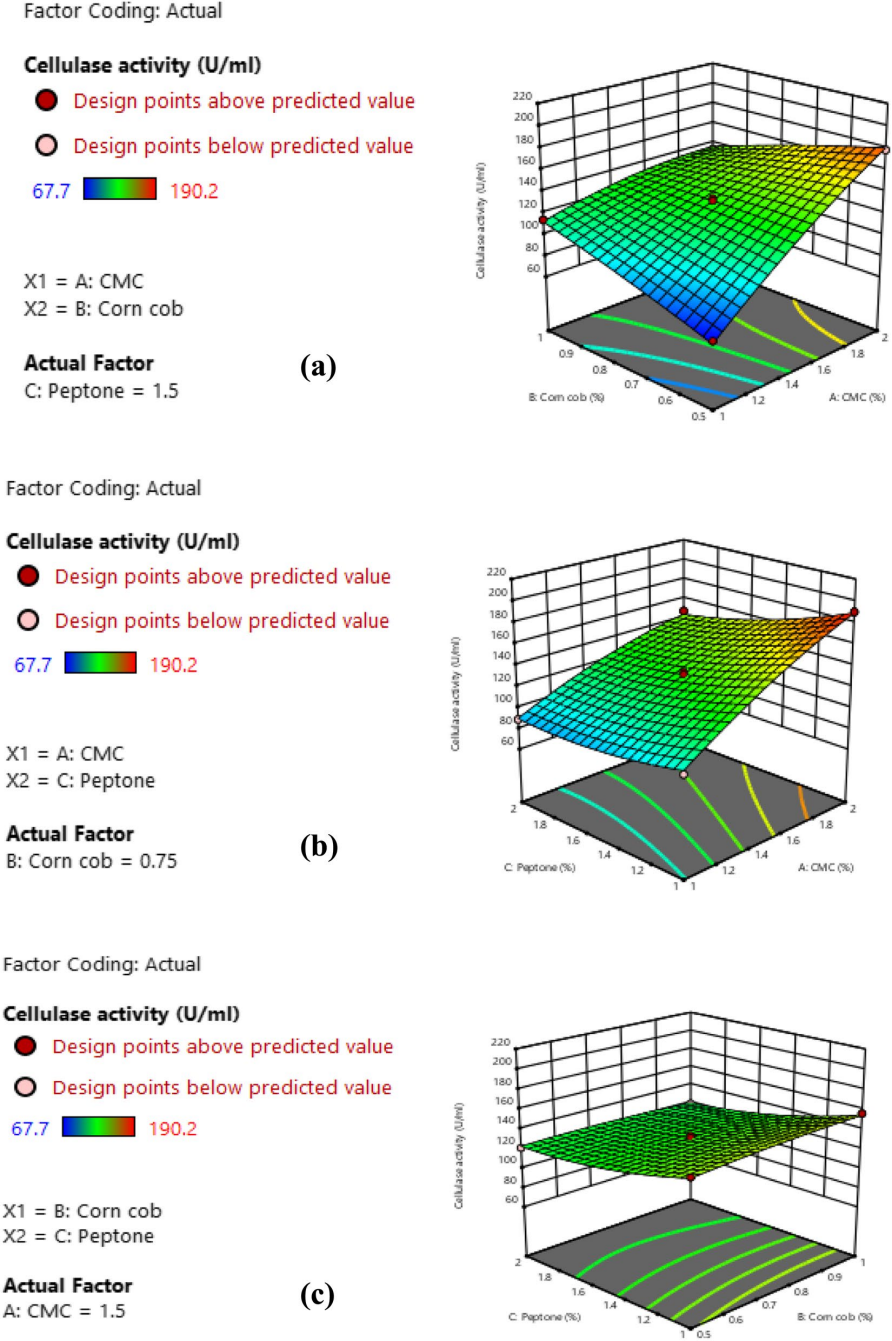
Response surface 3D graphs displaying the interaction among the tested variables that affect cellulase productivity (**a**) CMC and corn cob (**b**) CMC and peptone (**c**) Corn cob and peptone

The three-dimensional (3D) surface graphs showed a visible demonstration for the interaction between tested variables to determine their optimum levels that affect enzyme production. These graphs (Fig. 3) have been created via drawing enzyme activity on z-axis versus any 2 variables and preserving the other variables at their zero (central) level. Figure 3a displays the interaction impact of CMC and corn cob on cellulase productivity by *B. licheniformis* strain-MA1, keeping peptone at 1.5% (central level). The highest cellulase activity (178.5 ± 0.33 U/mL) was gained at 2% CMC (high level) and 0.5% corn cob (low level). Also, Fig. 3b shows interaction among CMC and peptone, while corn cob was preserved at 0.75% (central level). In such condition, the maximum cellulase activity (190.2 ± 0.31 U/mL) was recorded at 2% CMC (high level) and 1% peptone (low level). Moreover, Fig. 3c exhibits the influence of corn cob and peptone on cellulase yield preserving CMC at 1.5% (central level). The highest activity (156.4 ± 0.66 U/mL) was observed at 1% corn cob (high level) and 1% peptone (low level).

### Validation and confirmation of BBd model

**Fig. 4:**
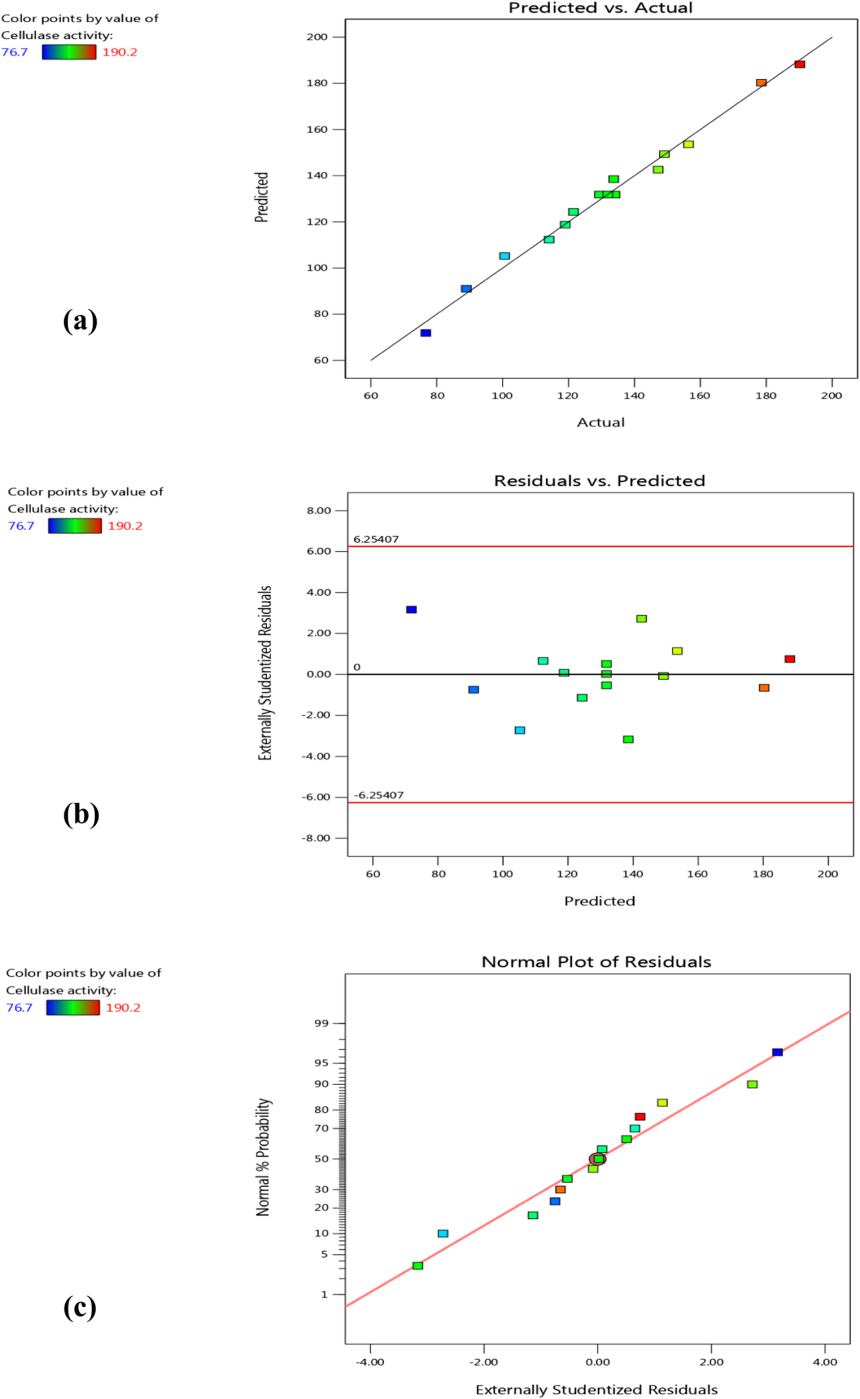
BBd for cellulase productivity by *B. licheniformis* strain-MA1 (**a**) The relation between predicted and actual values (**b**) Chart of residuals vs predicted values (**c**) Normal probability chart of the studentized residuals

Checking the validity of BBd model is very substantial to confirm that it provides adequate approximation to the real system. As shown in Fig. 4a, there was a high degree of accordance among predicted and actual values of cellulase activity, demonstrating the significance of BBd model and its validation to interpret the experiments. Also, the chart of residuals against predicted values shows the random scatter of residuals about a horizontal line (zero reference) which reflexes the adequacy of BBd to optimize cellulase productivity from *B. licheniformis* strain-MA1 (Fig. 4b). Otherwise, the normal probability chart of residuals exhibits the closure of the drawn points onto a straight line that indicates, the model is appropriate to the experimental data as displayed in Fig. 4c. For determining the model reliability and verifying the results, an experiment was carried out under the optimal levels predicted by the BBd to reach the desirability of cellulase activity and to get the highest enzyme production. The maximum cellulase activity predicted by the statistical model (BBd) using the optimum levels of the selected variables (CMC 2%, corn cob 0.5%, and peptone 1%) was 202.9 U/mL. Additionally, the experimental result that gave the highest cellulase activity (199.7 ± 0.34 U/mL) was in agreement with the predicted value by 98.4% which verifies the validation of the BBd model and the existence of optimal points. In the current study, BBd achieved the highest cellulase production (199.7 ± 0.34 U/mL) by *B. licheniformis* strain-MA1which is 6.8-fold more than that was obtained from the non-optimized medium. Similarly, cellulase productivity from *B. amyloliquefaciens* MBAA3 increased by 6.8-fold after applying statistical optimization [45]. On the other hand, Maravi and Kumar [46] reported that, medium optimization enhanced cellulase production from *B. licheniformis* by 2.5-fold compared to un-optimized media.

Finally, the optimized medium components using statistical designs (PBd and BBd) for cellulase production by *B. licheniformis* strain-MA1 were (g/L): CMC, 20.0; corn cob, 5.0; peptone, 10.0; (NH_4_)_2_SO_4_, 1.0; MgSO_4_.7H_2_O, 3.0; KH_2_PO_4_, 0.5; CaCl_2_, 1.0; at 30 °C for 72 h under 150 rpm and pH 7.0.

### Effect of *B. licheniformis* strain-MA1 cellulase on apple juice quality

#### Apple juice clarification

The effect of crude cellulase enzyme on physicochemical properties (yield, turbidity, apparent viscosity, TSS, total phenolic content as well as antioxidant activity) of apple juice are given in Table 5. The data showed a remarkable increase in juice yield in enzyme treated-juice sample (88.2 ± 0.15%) in comparison with control juice (75.4 ± 0.09%). This increase may be due to the release of water molecule and soluble solids after breaking cellulose polysaccharide by the enzyme during incubation time [47]. Also, the total soluble solids of clarified apple juice (9.86 ± 0.07) were somewhat higher than control juice (9.34 ± 0.08). The obtained results were in a good agreement with Handique et al. [48] when they used cellulase in banana juice production. The major type of fruit juices is the clear type compared to cloudy juice, the turbidity or cloudiness of apple juice is undesirable to most of consumers and considered as "muddy". The acceptability or rejection of fruit juice depends on consumer preference and type of juice like orange and tomato which accepted in the form of cloud [49]. Furthermore, the effect of cellulase on apple juice viscosity was shown in Table 5 that exhibited a significant difference (*P* < 0.05) between control and enzyme-juice treatment which was less viscous compared to control and more fluid. The reduction in viscosity after enzyme treatment may be due to cellulose hydrolysis by enzyme.

**Table 5:** Crude cellulase effect on some physicochemical properties of apple juice

Characteristics	Control	Enzyme-treated juice
Juice yield	75.4 ± 0.09^a^	88.2 ± 0.15^b^
Turbidity	3.95 ± 0.03^a^	0.47 ± 0.02^b^
Apparent viscosity (cp)	2.713 ± 0.09^a^	1.589 ± 0.05^b^
TSS	9.34 ± 0.08^a^	9.86 ± 0.07^a^
Total phenolic content (mg/mL)	0.957 ± 0.09^a^	0.412 ± 0.03^b^
Antioxidant activity (IC_50_)		
DPPH	56.8 ± 0.03^a^	82.7 ± 0.04^b^
FRAP	49.7 ± 0.06^a^	95.2 ± 0.07^b^

Values are expressed as mean ± SD

The apparent viscosity measured at shear rates of 1 to 437.4 s^−1^ of cloudy and enzyme treated apple juice samples and the obtained values are given in Table 5. It reaches about 2.713 ± 0.09 and 1.589 ± 0.05cp in control and enzyme treated apple juice, respectively at shear rate 437.4 s^−1^. The data in Table 5 illustrated the enzyme treatment caused a significant reduction in apparent viscosity (*P* < 0.05) of apple juice compared to cloudy sample. Moreover, the results in Table 5 revealed a significant (*P* < 0.05) decrease in total phenolic content in enzyme-treated juice sample which recorded (0.412 ± 0.03 mg/mL) only compared to control juice which had (0.957 ± 0.09 mg/mL). These findings were in accordance with Van der-Sluis et al. [50] who mentioned that processing steps during apple juice production had a dramatic effect on phenolic content reduction or loss in final product. An opposite trend had noticed in antioxidant activity which was increased significantly in control juice sample in comparison with enzyme-treated juice sample. The enzyme clarification treatment caused a reduction in antioxidant activity, and this may be due to the loss of cloud particle which contain the phenolic compounds especially that did not dissolve in water as mentioned by Bhushan et al. [51]; Oszmianski et al. [52] who found that, all clear and cloud apple juice samples contain gallic, chlorogenic acid and these compounds are highly soluble in water. Among the parameters used to measure the antioxidant activity is IC_50_ which expressed as the required concentration to obtain 50% radical scavenging of DPPH. The small values of IC_50_ represent the higher antioxidant activity. Therefore, the high values of IC_50_ in assays of DPPH or FRAP (Table 5) under the present conditions in clarified apple juice compared to cloudy juice correlated with the loss of phenolic compounds in particles after enzyme treatment. The reduction in total phenolic correlated with enzyme activity especially polyphenoloxidase (PPO) which degrade these compounds. The increase in antioxidant activity may be due to Maillard reaction compounds (MRPs) formation which cause increase in values of DPPH and FRAP tests.

#### Volatile compounds analysis

Twenty-seven volatile compounds were extracted using headspace solid-phase microextraction-gas and analysis was carried out by GC–MS and the obtained data were introduced in (Table 6) with available odor threshold values, as well as aroma description. The identified volatile constituents were belonging to several chemical classes: 15 esters; 6 alcohols; 4 aldehydes and 2 acids. These results in good agreement with published data on apple juice and apple products [53–55]. The most predominant class in apple juice volatile was esters (15 compounds) with sweet and fruity odor [56] followed by alcohols, particularly ethanol which represent about 17.25% and 16.37% in cloud and treated enzyme apple juice, respectively, as seen in Table 6. Also, the main esters in the present study were ethyl acetate (paint, fruity), ethyl propionate (fruity), hexyl butanoate and butyl acetate which had concentrations of 14.08%, 7.16%, 8.29% and 8.27% in cloud apple juice, respectively. While, the enzyme treatment caused an increase of ethyl acetate, ethyl propionate esters to be 16.34%, 8.43%, respectively and a decrease of butyl acetate (sweets, fruity) to be 7.62%. Butyl acetate and hexyl acetate were reported as the major esters in apple juice [57, 58]. Under our conditions for apple juice filtration all the aldehydes were decreased like hexanal and benzaldehyde or completely disappeared such as 2-methylbutanal except decanal which increased to be 2.35% in enzyme treated apple juice compared to control which had 1.94% (Table 6). The filtrated apple juice in this study recorded a decrease in the identified acids especially acetic acid which decreased from 2.05% in cloud apple juice compared to filtrated juice (1.63%) as shown in Table 6. The degradation products of fatty acids are alcohols which had higher odour threshold values compared to esters. So, they are considered the second major volatile compounds in apple juice [59]. In the present investigation the most predominant alcohols were ethanol in cloud and filtrate juices with concentrations of 17.25% and 12.38%, respectively. Likewise, the enzyme treated dapple juice showed a remarkable concentration of 1-butanol (3.05%) and 1-hexanol (2.86) compared to control sample which had 0.08% and 1.94% in the aforementioned alcohols, respectively (Table 6). The obtained results are in accordance with Nikfardjam and Maier [13]; Gan et al. [57] who reported that these alcohols play a domestic role in apple juice due to their sweet aroma properties especially 1-butanol. On the other side, acetic acid which represents about 66% of the volatile acidity in apple juice had reduced significantly after enzyme treatment. In both apple juice samples under investigation acetic acid was at normal level. However, there is need to look shade on the volatile distribution on cloud particles, serum and clear part of apple juice.

**Table 6:** Effect of *B. licheniformis* strain-MA1 cellulase on apple juice volatile compounds

Volatile compounds	LRI ^a^	Control	Enzyme-treated juice	OTH (μg/L)^c^	Odor descriptor ^d^
**Esters**					
Ethyl acetate	895	14.08^b^	16.34	5000	Paint, fruity
Ethyl propionate	961	7.16	8.43	10	Fruity
Propyl acetate	984	2.14	4.62		
Methyl butanoate	992	3.28	5.12	60	Fruity, cheese
Isobutyl acetate	1022	1.65	0.26		
Ethyl butanoate	1051	2.27	2.47	1	Fruity, apple
Propyl propionate	1058	1.95	5.34		
Ethyl-2-methyl butanoate	1066	4.68	3.25	0.006	Fruity, strawberry
Butyl acetate	1089	8.27	7.62	66	Sweets, fruity
2-Methylbutyl acetate	1134	1.63	0.86	5	Characteristic apple, banana like
Butyl butanoate	1153	3.26	1.29		Rotten apple
Ethyl hexanoate	1157	2.95	0.68		Fruity, green apple
Hexyl acetate	1176	4.12	5.94		Sweet fruity, floral
Hexyl butanoate	1185	8.29	9.25		Green
Hexyl hexanoate	1233	3.27	3.16		
**Alcohols**					
Ethanol	958	17.25	12.38	100,000	Sweet
2-Methyl-1-propanol	1107	0.62	0.02		
1-Butanol	1163	0.08	3.05	500	Overall flavour, sweet aroma
1-Hexanol	1367	1.94	2.86	150	Fresh, green, earthy
1-Octen-3-ol	1441	0.28	n.d		
1-Heptanol	1467	0.96	0.17		
**Aldehyde**					
2-Methylbutanal	944	0.16	n.d		
Hexanal	1098	2.05	1.64	5	Grass, tallow, fat
Decanal	1509	1.94	1.35		
Benzaldehyde	1534	1.62	0.82		
**Acids**					
Acetic acid	1447	2.05	1.63		Acid
Hexanoic acid	1815	1.04	0.92		

^a^Linear retention index

^b^Values are expressed as area percentage: n.d: not detected

^c^OTH: Odour threshold values cited from Anesea et al. [60]; Pino and Quijano[61]

^d^Odour description cited from[56, 62]

## Conclusion

The cellulolytic bacterial isolate, *B. licheniformis* strain-MA1 was obtained from soil sample of agricultural land in Egypt and its 16S rRNA gene sequence was recorded in the NCBI, GenBank database under accession number of ON840115. Cellulase production medium was optimized via multi-factorial designs (PBd followed by BBd). Optimization of medium parameters increased cellulase production by 6.8-fold comparing with the un-optimized one. The effect of *B. licheniformis* strain-MA1 crude cellulase on apple juice properties was evaluated. A remarkable increase in juice yield was noticed in enzyme treated-juice sample (88.2 ± 0.15%) compared to control juice (75.4 ± 0.09%). The total phenolic content in cloudy and clarified apple juice was 0.957 ± 0.09 and 0.412 ± 0.03 mg/mL, respectively. Also, DPPH and FRAP assays showed a remarkable increase in antioxidant activity (Low IC_50_) in control sample compared to enzyme treatment. Twenty-seven volatile compounds were extracted using headspace solid-phase microextraction-gas and analysis was done by GC–MS. The chemical classes of the identified volatile constituents including: 15 esters; 6 alcohols; 4 aldehydes and 2 acids. The predominant volatile class in apple juice was esters with sweet and fruity odor. Finally, the crude cellulase obtained from *B. licheniformis* strain-MA1 could be used as clarifying agent in apple juice.

## Supplementary Information

Supplementary Material 1

## Data Availability

The datasets generated and/or analyzed during the current study are available from the corresponding author on reasonable request. Additionally, the nucleotide sequence of 16S rRNA gene of the bacterial isolate Bacillus licheniformis strain-MA1 has been deposited in the NCBI, GenBank repository (https://www.ncbi.nlm.nih.gov/genbank/) under the accession number ON840115.
