# Supplementary figures and images for: Application of statistical designs strategy to improve cellulase production using agro-waste residue by a novel isolate Bacillus licheniformis strain-MA1 and assessing the enzyme effect on apple juice quality
Source: BMC Microbiol. 2024 Nov 29;24:511. doi: 10.1186/s12866-024-03656-z (PMC11605881; doi:10.1186/s12866-024-03656-z)

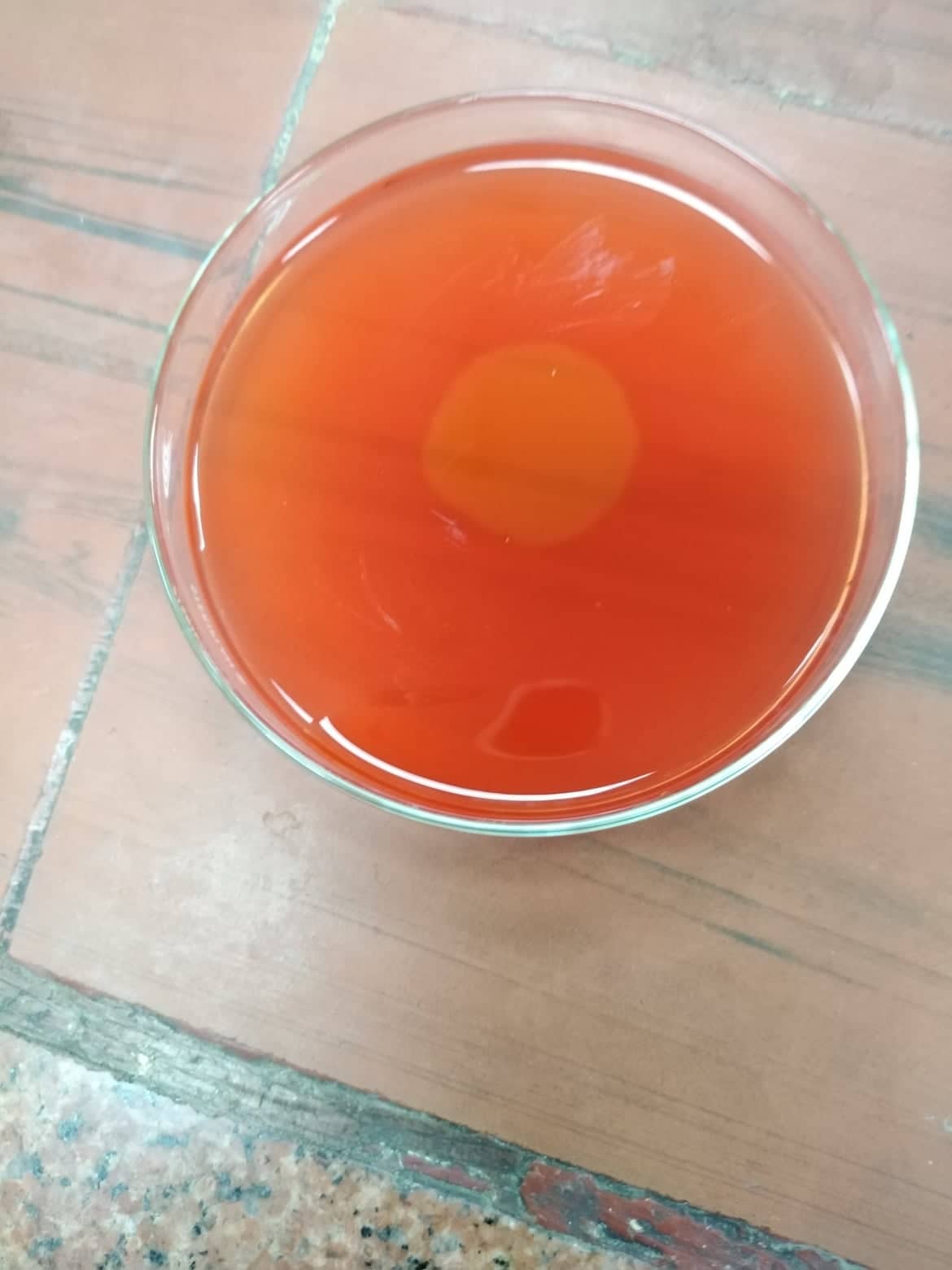


**Fig. S1** Qualitative screening of the bacterial isolate MA1 showing a clear zone on CMC-agar plate

Supplement: Supplementary file 1 — Supplementary Material 1 [file 12866_2024_3656_MOESM1_ESM.docx]
